# 

**Published:** 1870-08

**Authors:** 


### BOOK NOTICES.

"Life at Home," by WILLIAM AIKMAN, D. D., published by SAMUEL R. WELLS, 389 Broadway, New York, $1.50, is an admirable book, got up in excellent taste, relative to the family relation; with additional chapters about Husbands, Wives, Parents, Children, Brothers and Sisters, Employers and Employed, and the Altar in the House, or Family Prayer. The very subjects treated commend the book to multitudes of readers.

"Elm Island Stories," by Rev. ELIJAH KELLOGG, about "The Young Ship-builders," and a new novel for the family, "Life and Alone," are among the recent publications of LEE & SHEPARD, Boston, Mass., and afford pleasant and practical reading for the summer days, for the young particularly. $1.50 each.

"American Tract Society's 45th Annual Report, containing a list of the Auxiliaries and of the Life Directors and Members since the formation of the Society." Prosperity be to that great organization.

